# Human Arm Motion Prediction for Collision Avoidance in a Shared Workspace

**DOI:** 10.3390/s22186951

**Published:** 2022-09-14

**Authors:** Pu Zheng, Pierre-Brice Wieber, Junaid Baber, Olivier Aycard

**Affiliations:** 1 The Laboratoire d’Informatique de Grenoble, University of Grenoble Alpes, 38000 Grenoble, France; 2 Inria Centre at the University Grenoble Alpes, 38000 Grenoble, France

**Keywords:** human robot collaboration, human motion prediction, collision avoidance

## Abstract

Industry 4.0 transforms classical industrial systems into more human-centric and digitized systems. Close human–robot collaboration is becoming more frequent, which means security and efficiency issues need to be carefully considered. In this paper, we propose to equip robots with exteroceptive sensors and online motion generation so that the robot is able to perceive and predict human trajectories and react to the motion of the human in order to reduce the occurrence of the collisions. The dataset for training is generated in a real environment in which a human and a robot are sharing their workspace. An Encoder–Decoder based network is proposed to predict the human hand trajectories. A Model Predictive Control (MPC) framework is also proposed, which is able to plan a collision-free trajectory in the shared workspace based on this human motion prediction. The proposed framework is validated in a real environment that ensures collision free collaboration between humans and robots in a shared workspace.

## 1. Introduction

The third industrial revolution brought rapid progress to industrial automation and provided the solid foundation of modern manufacturing. During this era, many enterprise companies expanded and created a number of opportunities and businesses around the world. However, the main focus of this revolution was on automation of repetitive tasks in manufacturing industry and assembly lines [1]—for example, a robot doing a fixed task with hard-coded trajectories. The fourth industrial revolution is changing the life of every individual by altering the way of living. In this revolution, human and robots are working smartly together in shared environments and the the trajectories for robots are not hard-coded—these trajectories are dynamic or predicted using machine learning.

An important component of human capacity to interact with the world resides in the ability to predict its evolution over time. Handing an object to another person, playing sports, or simply walking in a crowded street would be extremely challenging without our understanding of how people move, and our ability to predict what they are likely to do in the following instants. Similarly, machines that are able to perceive and interact with moving people, either in physical or virtual environments, must have a notion of how people move. Since human motion is the result of both physical limitations (e.g., torque exerted by muscles, gravity, moment preservation) and the intentions of subjects (how to perform an intentional motion), motion modeling is a complex task that should be ideally learned from observations.

Collaborative robots, a.k.a cobots, offer a solution for small and medium-sized companies that require a flexible, fast, and precise operational solution in a shared workspace [2]. These cobots have proven to be intrinsically safe due to their ability to detect collisions and react accordingly [3]. However, it is always preferable to avoid collisions [4], and if a cobot is able to re-plan its trajectory to avoid collisions with a human in a shared workspace, there is an increase in productivity with respect to task completeness, effectiveness, safety, and throughput of human and cobot working time [5].

In our previous work, we proposed a model for predictive control scheme to generate trajectories for cobots that ensures a collision free environment in a shared workspace with humans by separating the work plane for the cobot [6]. The cobot automatically changes its trajectory to avoid the collision if a human hand enters the cobot plane. In this case, the collision can not be avoided, and the cobot ensures being at rest at the time of collision.

In this paper, we extend our previous work by predicting trajectories of human hands with our online trajectory generation framework [6] to ensure a safe and efficient human–robot collaboration in a shared workspace. The trajectories generated for cobots are fused with the predicted trajectories of a human hand to ensure a collision free environment. The perception module is composed of two parts: (1) detection and localization of the human hand in shared workspace, and (2) prediction of the human hand trajectory. Object detection from an RGB image is extensively studied since the development of deep learning libraries such as Openpose [7] and Mediapipe [8], which can achieve real-time skeleton detection of a human in a 2D images. However, the Cartesian 3D position of the human is necessary for the generation of the cobot trajectory. By using the RGB-D camera, we can re-project the 2D coordinates of the RGB image into Cartesian space.

This paper is organized as follows: Section 2 gives brief discussion on related work, Section 3 gives a formulation of our collision-free motion generator with a definition of collision avoidance constraints through separating planes, and a formulation through Quadratic Programs (QP). Section 4 explains the detection of the human pose from a single monocular 3D camera and the projection into Cartesian space. The prediction model is explained in Section 5. Experiments and results are discussed in Section 6; finally, conclusions and future work are discussed in Section 7.

## 2. Related Work

Our work mainly contributes to human robot collaboration (HRC) using human motion prediction, particularly the human hand, in a shared workspace with a cobot. There are multiple ways and practices to detect the human for tracking and prediction in HRC systems [9,10,11,12]. Use of monocular camera, depth camera, 3D LiDAR, and inertial measurement unit (IMU) sensors for motion capture is a common practice. The IMU sensor based methods are not suitable for collaborative tasks, and the 3D LiDARs are relatively expensive in terms of cost and computation. However, using the vision sensors such as RGB cameras is a widely used approach for human motion prediction [9]. The main limitation of RGB cameras includes a range of camera sensors and enabling 3D human position w.r.t the robot. Using an RGB-D camera, which is a combination of a single view from monocular and depth sensors, provides a balance between simplicity and performance [13].

The depth camera based on time-of-flight (TOF) and structured light technologies can provide the distance information from a single depth image; thus, it creates the possibilities to deal directly with 3D data [14]. Moreover, human pose estimation has some unique characteristics and challenges such as flexible body configuration indicating complex interdependent joints, diverse body appearance, and complex environment may cause occlusion. The existing approaches can be divided into three categories: template-based method, feature-based method, and learning-based method [15]. The template-based method compares the similarity between the detected object and the constructed template to identify the motion category [16,17]. The template-based methods need to establish a template library of parameterised template to compare with the human body, which is time-consuming, and the accuracy of template-based methods is very limited due to the diversity of the different human pose in space. Feature-based methods use geodesic distance information [18], geometric features such as silhouette [19], to estimate the human joints. The feature-based template has some disadvantages; for instance, it requires prior knowledge to combine with extracted global or local features to obtain the 3D pose, and it is not suitable for changing poses.The learning-based method use the network structure to automatically learn the required features from input data. The learned features can be further used to extract the human poses [20].

Recently, deep learning based models are getting popular [11,21,22,23,24]. Deep learning methods can take unprocessed data such as point clouds and produce human poses with high accuracy [24]. However, deep learning models are data greedy models and require heavy data for training which are not easily available for human hand prediction, and datasets contain point clouds that are not suitable for real-time applications. Therefore, as a more accessible approach, estimating human poses from RGB images captured by regular cameras and then mapping the 2D information into 3D space is efficient and widely practiced in industry and academia [6,9].

The trivial way to estimate 3D human poses is to design an end-to-end network to predict the 3D key-point locations directly from 2D images [25,26]. However, recovering a 3D human pose from a single image is still challenging and leads to many false positives. Using depth information from RGB-D cameras can effectively transform 2D pose location into 3D [6].

Existing human motion prediction methods can generally be divided into model-based and learning-based approaches. Model-based approaches attempt to directly model the kinematics or dynamics of the human and thus find the corresponding arm motor control [27], and human movements follow an optimal feedback control strategy that connects together motor behaviour, limb mechanics, and neural control. The detailed description on human arm motor control can be found in [27]. However, the choice of optimal trajectory cost is not trivial because the human musculoskeletal system presents more degrees of freedom (DoF). This kinematic, dynamic, and actuation redundancy issue is not straightforward in terms of motion equations. Numerous cost functions have been identified in literature [28,29,30,31]. In [28], the authors model the hand’s point-to-point kinematic motions with minimum Cartesian jerks (third derivative of Cartesian coordinates) for an arm movement in the horizontal plane. Authors in [29] incorporate dynamics with a minimum torque change model in the horizontal plane; however, the results are not validated for 3D movements. While defining these motor control criteria manually is difficult, the author in [30] defines a combination of seven different criteria (such as Cartesian jerk, angle jerk, angle acceleration, torque change, torque, geodesic and energy) and an inverse optimisation method has been used to find the weight associated with each criteria. In [31], instead of finding arm motor control artificially, the authors over-approximate the occupancy of the arm with a maximum velocity model, but this can be too restrictive if the prediction horizon is long. These approaches have several limitations such as the dynamics of the human being highly nonlinear and non-deterministic; it can vary according to emotions and physical condition, so direct modelling can be quite inaccurate in different situations. Moreover, the hypothesis about the human’s rationality is often invalid; hence, constructing an optimisation criteria based on this hypothesis can be very ambiguous, and the combination of the different criteria is chosen manually.

Human motion is the result of complex bio-mechanical processes that are challenging to model. As a consequence, state-of-the-art work on motion prediction focuses on data-driven models [9,32,33,34,35,36,37] such as probabilistic models [35,36] and deep learning models [9,37]. Recent work on short-term human motion prediction has centered on a Recurrent Neural Network (RNN) due to their capacities to handle sequential data. The RNN can remember important things about the input received that allow them to be very accurate in predicting the output. In the proposed framework, an RNN based approach is used to predict the human hand motion which is then passed to our motion planning package for collision avoidance with the cobot.

## 3. General Trajectory Planning Scheme

**Figure 1:**
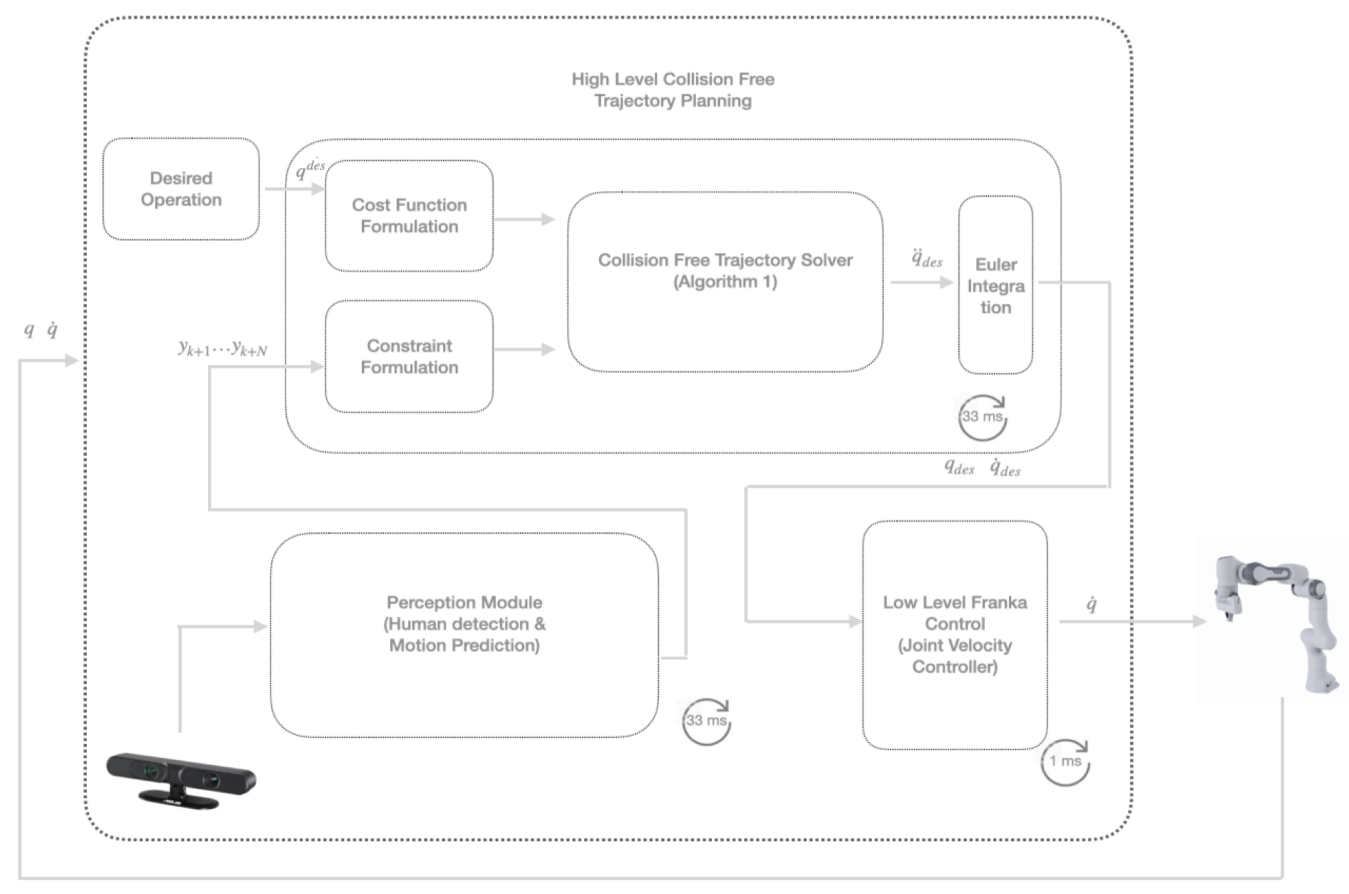
Abstract flow diagram of control architecture.

The goal of our scheme is to generate a collision-free trajectory for a cobot (e.g., a 7-DoF manipulator cobot) that has to perform a task in a workspace shared with a human worker. This scheme is composed of three parts, as shown in Figure 1: (i) a human motion prediction module, (ii) a collision-free trajectory generation module, and (iii) a low-level robot motion control module.

In this section, we summarise how to compute a collision-free trajectory based on the perception module and the cobot’s dynamics. In addition, we emphasise the role of the terminal constraint to guarantee safety. This terminal constraint provides a *passive motion safety* guarantee [38], which means that, if a collision occurs, the cobot is at rest at the time of the collision so that it does not inject its own kinetic energy. In the following, we recall the main equations from the MPC approach developed in our previous work [6].

### 3.1. Separating Plane Optimisation

**Figure 2:**
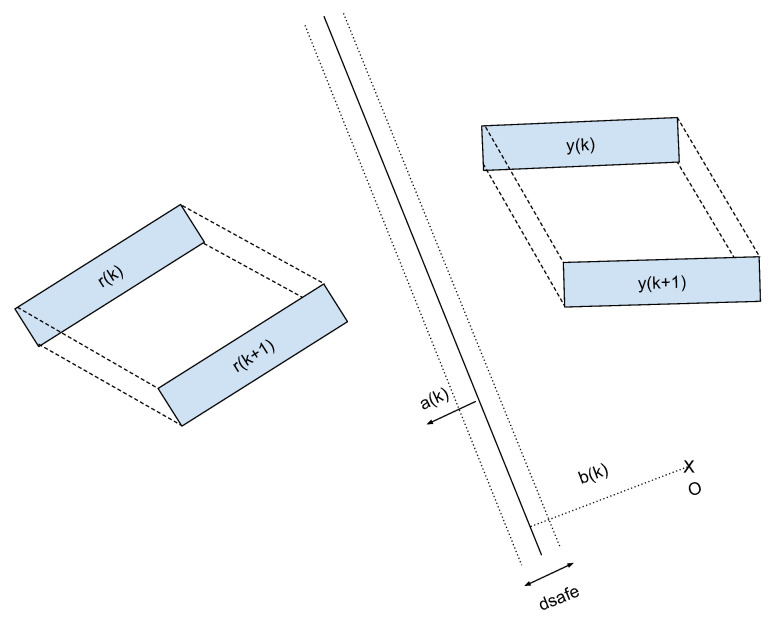
An illustration of separating plane between two objects.

As illustrated in Figure 2, if there exists at the prediction time k∈N a plane defined by a normal vector $a_{k}\in R^{3}$ and a scalar constant $b_{k}\in R$ such that all vertices $y^{j}$ related to the human stay on one side between instants *k* and k+1 while all vertices $r^{i}$ related to the cobot stay on the other side, then we have evidence that they do not collide over this interval of time. Here, $j\in \{1,\ldots N_{p}\}$ and $i\in \{1,\ldots N_{r}\}$ where $N_{p}$ and $N_{r}$ are the number of vertices associated with the human and the cobot, as appears in the constraints, Equations (1b)–(1e), and the distance d∈R controls the position of the separating plane between the human and the cobot.

(1a) $$\begin{array}{cc} \underset{a_{k},\;b_{k},\;d}{\min.}\;\; & -d+\alpha d^{2}+\beta ∥a_{k}-a_{k}^{p}{∥}^{2}+\beta ||b_{k}-b_{k}^{p}{\left|\right|}^{2} \end{array}$$

(1b) $$\begin{array}{cc} \mathrm{s.t.}\;\; & \forall \;j\in \left\{1,\ldots N_{p}\right\},\;a_{k}^{T}y_{k}^{j}\leq b_{k}, \end{array}$$

(1c) $$\begin{array}{cc} \; & \forall \;j\in \left\{1,\ldots N_{p}\right\},\;a_{k}^{T}y_{k+1}^{j}\leq b_{k}, \end{array}$$

(1d) $$\begin{array}{cc} \; & \forall \;i\in \left\{1,\ldots N_{r}\right\},\;a_{k}^{T}r_{k}^{i}\geq b_{k}+d, \end{array}$$

(1e) $$\begin{array}{cc} \; & \forall \;i\in \left\{1,\ldots N_{r}\right\},\;a_{k}^{T}r_{k+1}^{i}\geq b_{k}+d, \end{array}$$

(1f) $$\begin{array}{cc} \; & -1_{3}\leq a_{k}\leq 1_{3}, \end{array}$$

(1g) $$\begin{array}{cc} \; & 1-\varepsilon \leq a_{k}^{T}\;a_{k}^{p}\leq 1 \end{array}$$

We want to maximise the distance *d* between the separating plane and the cobot. Given the formulation as a minimisation problem, we include the term −d in the cost function, Equation (1a). The following term in the cost function smooths the variations of separating planes, with $a_{k}^{p}$ and $b_{k}^{p}$ the separating plane parameters obtained at the previous sampling time, and some small weights α and β. Finally, we use constraints, Equations (1f) and (1g), to approximate a nonlinear constraint to bound the vector $a_{k}$ to a unit norm, where $1_{3}\in R^{3}$ is a row vector of ones.

### 3.2. Optimal Motion Generation

Once we have a sequence of separating planes parameters, we can include them in our MPC scheme to compute an optimal collision-free trajectory:

(2a) $$\begin{array}{cc} \underset{u}{\min.}\;\;\; & \sum_{k=0}^{N-1}∥s_{k+1}-s_{k+1}^{\mathrm{des}}{∥}_{Q}^{2}+∥u_{k}-u_{k}^{\mathrm{des}}{\left|\right|}_{R}^{2} \end{array}$$

(2b) $$\begin{array}{cc} \mathrm{s.t.}\;\; & \forall \;k\in \left\{0,\ldots N-1\right\},\;\underline{u}\leq u_{k}\leq \bar{u}, \end{array}$$

(2c) $$\begin{array}{cc} \; & \forall \;k\in \left\{1,\ldots N\right\},\;\underline{q}\leq q_{k}\leq \bar{q}, \end{array}$$

(2d) $$\begin{array}{cc} \; & \forall \;k\in \left\{1,\ldots N-1\right\},\;\underline{\dot{q}}\leq {\dot{q}}_{k}\leq \bar{\dot{q}}, \end{array}$$

(2e) $$\begin{array}{cc} \; & {\dot{q}}_{N}=0,\\ \; & \forall \;k\in \{0,\ldots N-1\},\;\forall \;i, \end{array}$$

(2f) $$\begin{array}{cc} \; & \;\;\;\;\;a_{k}^{T}r_{k}^{i}\left(q_{k}^{p}\right)+a_{k}^{T}J\left(q_{k}^{p}\right)\left(q_{k}-q_{k}^{p}\right)\geq b_{k}+d_{\mathrm{safe}},\\ \; & \forall \;k\in \{0,\ldots N-1\},\;\forall \;i, \end{array}$$

(2g) $$\begin{array}{cc} \; & a_{k}^{T}r_{k}^{i}\left(q_{k+1}^{p}\right)+a_{k}^{T}J\left(q_{k+1}^{p}\right)\left(q_{k+1}-q_{k+1}^{p}\right)\geq b_{k}+d_{\mathrm{safe}} \end{array}$$

where $q_{k}\in R^{n}$ and ${\dot{q}}_{k}\in R^{n}$ are respectively the joint position and velocity, with *n* the number of degrees of freedom. The state $s_{k}\in R^{2n}$ includes $q_{k}$ and ${\dot{q}}_{k}$, and $u_{k}\in R^{n}$ is the control input (acceleration) of the cobot.

Our prediction horizon has a length N∈N. The cost function, Equation (2a), is designed to track a desired joint state trajectory $q_{k}^{des}$ with acceleration $u_{k}^{des}$, while $\underline{q}$, $\bar{q}$, $\underline{\dot{q}}$, $\bar{\dot{q}}$, $\underline{u}$, $\bar{u}$ indicate minimum and maximum joint positions, speed, and acceleration (we assume that $\underline{\dot{q}}\leq 0\leq \bar{\dot{q}}$ and $\underline{u}\leq 0\leq \bar{u}$). The terminal constraint, Equation (2e), ensures that the cobot is at rest at the end of the prediction horizon in order to provide a passive motion safety guarantee, making sure that the cobot is able to stop and stay at rest before any collision happens in the future. Equations (2f) and (2g) introduce the collision avoidance constraint based on separating planes, which is computed by linearising the kinematics of the cobot around the previously computed trajectory:

(3) $$r_{k}^{i}=r^{i}\left(q_{k}\right)\approx r^{i}\left(q_{k}^{p}\right)+J\left(q_{k}^{p}\right)\left(q_{k}-q_{k}^{p}\right)$$

## 4. Human Pose Detection

**Figure 3:**
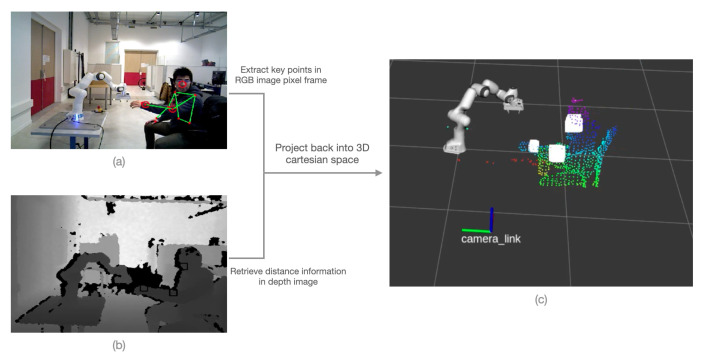
Example demonstration of RGB-D image for mapping human hand in 3D space, (**a**) shows RGB image on which hand joins are detected, (**b**) shows the bounding box around the points in the depth image, and (**c**) shows the mapping of points in 3D space.

The Algorithm 1 gives the set of instructions for collision free trajectory computation. In this section, we explain how to detect a human’s position and train the model for prediction.
**Algorithm 1:** Collision free trajectory computation.Input: $U_{k}^{p}$, $S_{k}$, $a_{k}^{p}$, $b_{k}^{p}$Output: $U_{k}$1:i = 0;2:**while** ($\left|\right|U_{k}-U_{k}^{p}{\left|\right|}^{2}$ OR i≤k) **do**3:    $U_{k}^{p}=U_{k}$;4:    /* Updating Robot Parameters */5:    {a,b} ⟵ Solve Equation (1) for k∈{0,…N−1};6:    {$U_{k}$} ⟵ Solve Equation (2);7:    i ++;

The perception system used in this work is an ASUS Xtion Pro depth camera which provides cloud points, colour and depth images. We can then recover the (x,y,z) position in Cartesian space by combining color and depth image information. The advantage of working with 2D images is that we can directly use existing deep learning libraries such as OpenPose [39] or MediaPipe [8], which are fast and robust. Mediapipe is widely used for several real-time applications such as tracking [40], and sign language understanding [41]. An example of human upper-body key points extraction is shown in Figure 3a. With the lightweight version of this deep learning model, the inference speed is performing at 0.25 s on a MacBook Pro (2017).

After obtaining the coordinates of the joints in the colour image frame, we can map the corresponding coordinates in the depth image to find the distance between the camera and the pixel points. This mapping necessitates a proper calibration of the camera [42]. The distance information allows us to compute the key point’s Cartesian location using the pinhole camera projection model, since we know the camera’s intrinsic parameters:

(4) $$\begin{array}{cc} X & =\left(u-c_{x}\right)\frac{Z}{f_{x}} \end{array}$$

(5) $$\begin{array}{cc} Y & =\left(v-c_{y}\right)\frac{Z}{f_{y}} \end{array}$$

(6) $$\begin{array}{cc} Z & =Z \end{array}$$

where *u* and *v* are key point locations in pixel coordinates, $c_{x}$ and $c_{y}$ are camera offset, $f_{x}$ and $f_{y}$ are camera focal parameters, and Z is the distance given by the depth image.

The information contained in the depth image is sensitive to disturbances and background elements. To make the result more robust and accurate, we define a bounding box around the key point to eliminate outliers and average the distances, as shown in Figure 3b. Finally, we successfully map the key point’s from RGB image to 3D location, as shown in Figure 3c.

## 5. Human Hand Motion Prediction

**Figure 4:**
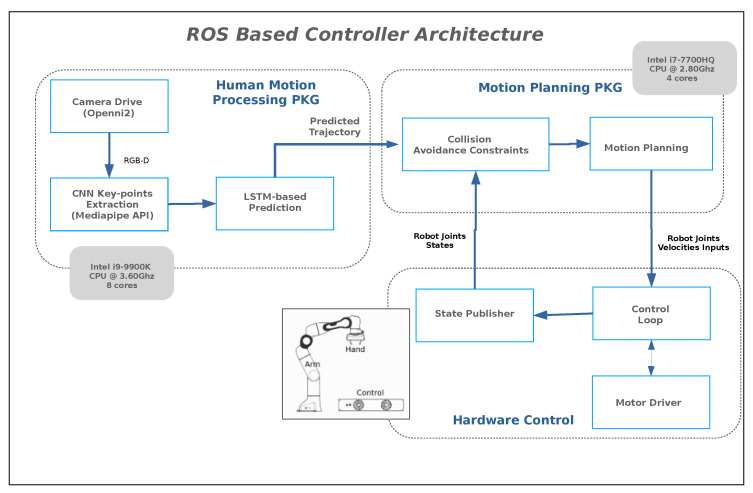
ROS based controller architecture to enable collaboration between humans and robots in shared environments.

Figure 4 shows the overall architecture of human hand prediction, robot controlling, and collision avoidance. It has basically three modules; in the first module, it extracts the human hand trajectory from the RGB-D camera and passes to our trained prediction model: in the second module, the predicted trajectories are handled by a motion planning package to detect and avoid collision: finally, the last module controls the motion of the robot based on the feedback by a motion planning module. The configuration for PC used is also shown in Figure 4; the average time to extract hand trajectory and prediction is 0.04 s, whereas the frame per seconds (fps) from RGB-D camera is 33, which makes our prediction real-time on commodity hardware. The 0.04 seconds comprise time to extract CNN based keypoints for hand motion trajectory generation and LSTM based prediction. The motion planning package takes 0.01 seconds/frame to ensure collision avoidance. The simulation demo of human and cobot collaboration on shared workspace can be seen online (https://www.youtube.com/watch?v=PAZZRtS7Qc4, accessed on 17 August 2022).

**Figure 5:**
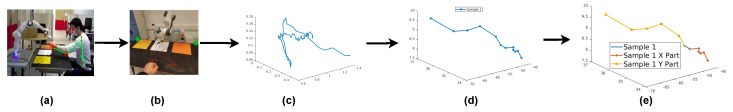
Demonstration of the environment for dataset generation, (**a**) shows the person working in shared environment with a cobot, (**b**) shows the possible goals on which the hand should be moving, (**c**) shows one sample hand motion trajectory generated over one minute, (**d**) shows the sub-trajectory over 12 observations, and (**e**) shows the trajectory divided into two sequences (*x* for training and *y* for prediction).

The dynamics of the human can be described in state-space from Equations (7a) and (7b). Without loss of generality, we consider only the dynamics of a human’s hand position to simplify notations:

(7a) $$\begin{array}{cc} x_{t+1} & =g(x_{t},w_{t}) \end{array}$$

(7b) $$\begin{array}{cc} y_{t} & =h(x_{t}) \end{array}$$

where $x_{t}\in R^{3}$ is the discrete time variable describing the human’s hand position, $w_{t}\in R^{3}$ is the muscular force or external effect that causes the human’s movement, which is not known; the function *g* represents the human’s hand dynamics, and $h_{t}\in R^{3}$ is the measurable position given a state $x_{t}$. We assume that the movement of the human is not completely random and follows patterns as shown in Figure 5b, where dotted red lines denote representative motions to different goals.

In order to anticipate the human’s future motion, it’s not sufficient to predict only one-step ahead as shown in Equation (7a). In a more general scenario, we want to predict *T* steps ahead given a current state $x_{t}$ and $w_{t}$ and consider L-order Markov assumptions. Therefore, we can formulate this problem as: given a time-series input **x** = $\left\{x_{t},x_{t-1},\cdots ,x_{t-L}\right\}$, we want to find a function ϕ such that: ϕ:x→y, where L is the number of past observations and **y** = $\left\{x_{t+1},x_{t+2},\cdots ,x_{t+T}\right\}$ with T the number of steps to predict.

**Figure 6:**
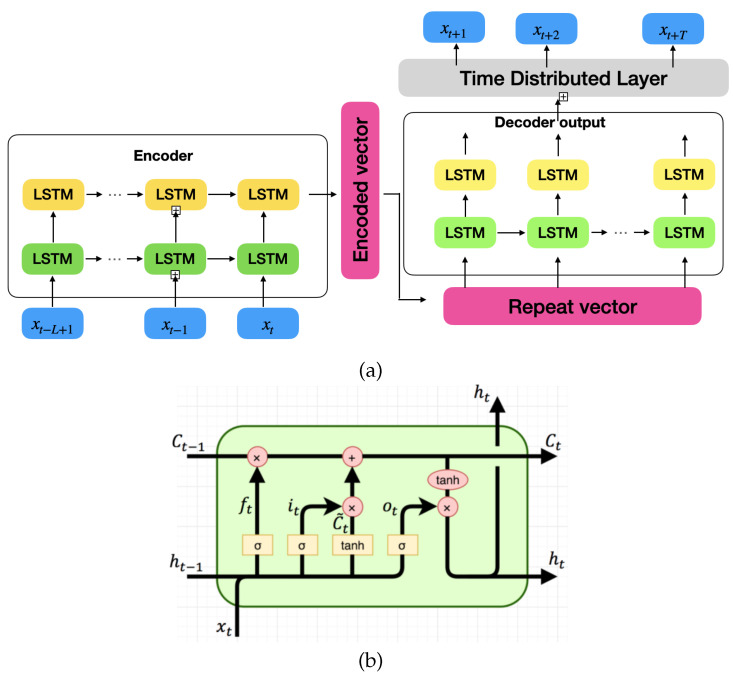
Proposed model for human hand motion prediction, (**a**) shows the architecture of the encoder–decoder LSTM neural network, and (**b**) shows the overview of LSTM cell architecture.

Modelling such a dynamics function is very challenging because the external factors are not measurable and unpredictable. Moreover, the dynamics of the human are highly nonlinear. However, neural network structure is efficient to learn such nonlinear mapping patterns. We define our prediction network structure in Figure 6a as an encoder–decoder model. The past observation data are encoded through several stacked Long Short-Term Memory (LSTM) layers to increase the depth of the network. The encoded information is passed into an LSTM decoder layer followed by a fully connected layer to produce the final multi-step prediction. The structure of an LSTM cell is shown in Figure 6b and the mathematical formulation is as follows:

(8a) $$\begin{array}{cc} f_{t} & =\sigma (W_{f}\cdot \left[h_{t-1},x_{t}\right]+b_{f}) \end{array}$$

(8b) $$\begin{array}{cc} i_{t} & =\sigma (W_{i}\cdot \left[h_{t-1},x_{t}\right]+b_{i}) \end{array}$$

(8c) $$\begin{array}{cc} \tilde{C_{t}} & =tanh(W_{C}\cdot \left[h_{t},x_{t}\right]+b_{C}) \end{array}$$

(8d) $$\begin{array}{cc} C_{t} & =f_{t}*C_{t-1}+i_{t}*\tilde{C_{t}} \end{array}$$

(8e) $$\begin{array}{cc} o_{t} & =\sigma (W_{o}\cdot \left[h_{t-1},x_{t}+b_{o}\right] \end{array}$$

(8f) $$\begin{array}{cc} h_{t} & =o_{t}*tanh\left(Ct\right) \end{array}$$

where $\left[W_{f},b_{f}\right]$, $\left[W_{i},b_{i}\right]$, $\left[W_{C},b_{C}\right]$ and $\left[W_{o},b_{o}\right]$ are learn-able weights and bias, $f_{t}$ and $i_{t}$ are forget gate and update gate, and $h_{t-1}$ and $h_{t}$ are previous and current hidden states, respectively. $\tilde{C_{t}}$ is the new candidate cell value. Thus, the new cell state $C_{t}$ is updated by $C_{t-1}$ and $\tilde{C_{t}}$ with associated forgetting weight and update weight. The new output and new hidden state are represented by $o_{t}$ and $h_{t}$. In the end, the predicted positions are provided to the separating plane computation to formulate constraints Equations (1b) and (1c).

## 6. Experiments and Results

As stated in the Introduction, the collision avoidance by predicting human motion prediction has potential applications in the industry. However, there is still no benchmark dataset for experimentation and learning. Therefore, very few works have been reported. In this paper, we generate a dataset with real cobot coordination. The Franka Emika Panda cobot is used for the experimentation. In our previous work, we used a similar cobot for collision detection [6]. To generate the human hand trajectories, a human is asked to perform some tasks on a shared workspace with the cobot. The human hand moves to several different goals with some patterns with different speed and position; the patterns on which the hand should be moved is shown in Figure 5b, and the overall flow diagram for dataset generation is shown in Figure 5.

Let the learning data of K observation sequences be collected from the RGB-D camera, as demonstrated in Figure 5, S = $\left\{S^{1},\cdots ,S^{K}\right\}$, where $S^{k}=\left\{S_{1}^{k},\cdots ,S_{T_{k}}^{k}\right\}$. Each element $S_{t_{k}}^{k}$ denotes the position of the hand in Cartesian space. In this experimentation, the human’s hand moves to several different goals with some patterns shown in Figure 5b. For each task, we generate five similar trajectories with minor changes in position and speed. Raw trajectory data are shown in Figure 5 (a partial trajectory), which can be used with relative positions for better learning [43].

We transform the absolute coordinates of the hand’s position into relative coordinates (relative displacements) to let coordinates become scene independent. In this way, the model will learn the motion displacement pattern instead of memorizing the trajectory. Secondly, we apply the data augmentation method to increase model generalisation capability. We use random rotation to each trajectory to make the network learn rotation-invariant patterns. We add Gaussian noise with mean 0 and small standard deviation to every point to make the network more robust to small perturbations and imprecision. Furthermore, we divide these trajectories into prediction windows. For example, we define one training sample as χ=(x,y) with *x* and *y* tensor of shape two corresponding to time-step and features size, as shown in Figure 5e.

We demonstrate the proposed optimal collision-free trajectory planner with a 7-DoF manipulator cobot. Maximum joint speed and acceleration are respectively $\frac{\pi }{2}$ rad.s${}^{-1}$ and 10 rad.s${}^{-2}$. We opt for a prediction horizon of length 0.25 s, with sampling time Δt = 0.05 s and N = 5, which covers the time necessary for the cobot to stop completely under all circumstances, in order to satisfy the terminal constraint, Equation (2e), and enable in this way the passive motion safety guarantee. A longer prediction time could provide improved collision avoidance, but this would be highly dependent on the precision of longer-term human motion prediction.

**Figure 7:**
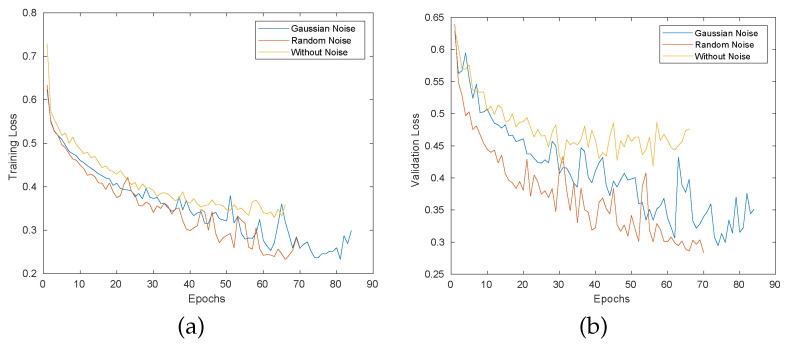
Training and validation loss of proposed model, (**a**) shows the training and validation MAE for the model with Gaussian noise, and (**b**) shows the training and validation MAE with random noise.

The safety distance is chosen equal to $d_{\mathrm{safe}}=$ 20 cm. The cobot completes a pick-and-place task between positions $G_{rA}=$ (0.5, 0.4, 0.2) m and $G_{rB}=$ (0.5, −0.4, 0.2) m expressed in the frame of the cobot base link. These two positions are shown as the green balls in Figure 5b. In addition, the human moves his hand following the trajectory patterns shown in Figure 5b. The neural network model is implemented in Tensorflow [44], and the total network parameters are 149,699. The encoder part consists of three stacked LSTM layers with 64 units for each layer. We add $l_{1}$ and $l_{2}$ regularisation with weights 1 $\times 10^{-4}$ and 1 $\times \;10^{-5}$, respectively. The decoder has the same structure as the encoder, and we change the final output layer by a time distributed by using fully connected layers to predict future relative displacements. The MSE (mean squared error) is used as loss function for our deep learning model, and the original data are augmented by adding random rotations and Gaussian noise, as explained above. The Gaussian noise is widely used for data augmentation. However, we experimented with Gaussian noise, random noise, and without any kind of noise. Figure 7 shows the learning loss over different epochs, (a) shows training loss, and (b) shows validation loss.

**Figure 8:**
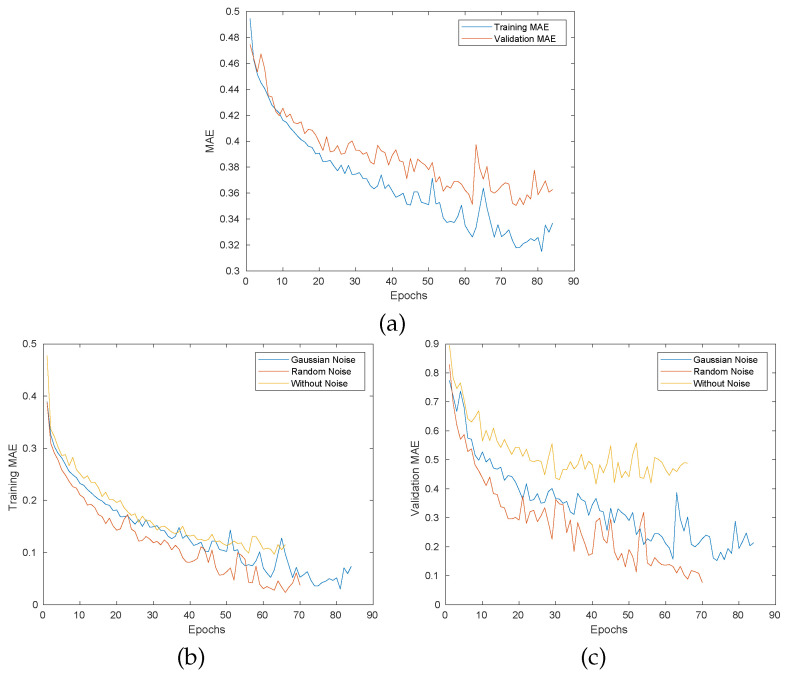
The MAE of proposed model, (**a**) shows the training and validation MAE for the model with Gaussian noise, (**b**,**c**) show the comparative validation and training MAE with a different configuration of noise.

The model is evaluated based on MAE (mean absolute error), which is a widely used metric for evaluating the prediction based models [45,46,47,48]. The MAE is basically the sum of absolute difference between ground-truth 3D position and predicted position. Figure 8 shows the MAE for the proposed model; (a) shows the MAE for validation and training set with Gaussian noise; (b) and (c) show comparative curves for random noise and models without noise. It can be seen that the model without noise has a high error on the validation set, which clearly gives the impression of over-fitting. Surprisingly, the random noise gives an overall minimum error on the training and validation set, but the Gaussian based model works better on a cobot when these models were deployed for real-time testing.

**Figure 9:**
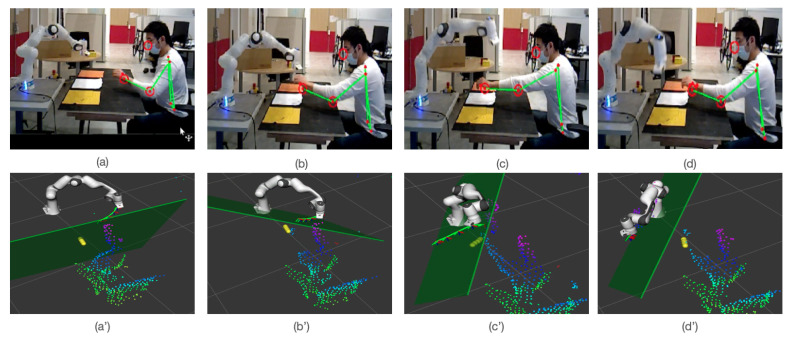
The qualitative visualization of collaborative environment between cobot and human, (**a**) shows the distance between cobot and hand, which is much larger than safety distance (20 cm), (**b**) shows the behavior of the cobot when the human hand is intentionally placed for possible collision, the cobot deviates from its initial trajectory to avoid the collision, (**c**,**d**) show that the cobot achieves its goal without stopping or hurting the human while keeping a safe distance. The sub-figures from (**a’**–**d’**) show the corresponding visualization of the same data in RVIZ.

The qualitative visualization of deployed model is shown in Figure 9. In Figure 9a,a’, the cobots move from goal $G_{rA}$ to $G_{rB}$, and the collision-free trajectory is shown as successive frames in green. As the distance between the cobot and human is large enough, this trajectory is straight to the goal. The yellow spheres represent the predicted positions of the human’s hand in five time-steps. The green plane represents the separating plane (only the first predicted step is shown here, we have in total (N-1) planes). In Figure 9b,c and Figure 9b’,c’, the cobot deviates its trajectory in order to avoid the human motion. The predicted positions are shown according to the yellow sphere. Finally, the cobot attends the position of $G_{rB}$ with successful collision avoidance, as shown in Figure 9d,d’.

**Figure 10:**
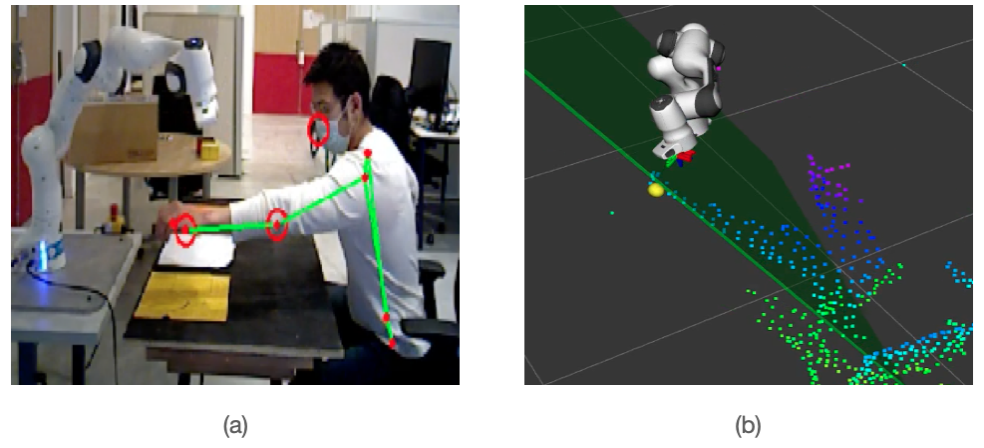
A case study on which collision can not be avoided, (**a**) human blocks cobot motion intentionally, (**b**) the trajectory generator ensures that the cobot is at rest to avoid collision.

To ensure human safety in a collaborative shared workspace, we evaluated different cases on which we ensured that the cobot achieves his goal by avoiding the possible collision with humans, as explained in previous experiments, and we also tested a situation which created a deadlock, and it is not achievable for the cobot to complete the task. Figure 10 shows the simulation on which the human hand is placed for longer duration, which creates no exception for completing the task. In this case, the motion generator keeps the cobot still at the desired safe distance.

The proposed MPC modules give competitive performance for various industrial tasks in shared environments. However, the proposed model ensures human safety and collision avoidance only if one hand is used. It is obvious that the perception module can not see the second hand of the human due to occlusion. The limitation of the proposed module can easily be removed by installing more than one RGB-D cameras to ensure the occlusion free perception.

## 7. Conclusions and Future Work

In this paper, we integrated a perception module into our previous safe MPC scheme to generate optimal collision-free trajectories online. In our previous MPC module, we detected the collision and ensured that, during collision, the cobot is at rest. In this work, we extended our previous MPC scheme; instead of detecting collision, we aimed to prevent it by predicting the human hand motion. Based on the prediction trajectories, the cobot changes its motion and maintains a safe distance from the human hand. Taking into account the future motion of a human’s hand can significantly help the motion generator to plan a collision-free trajectory. In this case, the task of the cobot is interrupted intentionally by the human, and MPC can not generate a collision free trajectory; then, the motion generator lets the cobot wait at a safe distance. However, using one camera leads to a problem of occlusion. In this case, the human second hand position can not be detected reliably even with state-of-the art algorithms. Our next goal is to extend our perception module with multiple cameras to ensure occlusion-free perception to our MPC. Furthermore, we want to generalize the hand’s prediction task to whole-body motion prediction where humans can work with autonomous mobile robots. 

## Data Availability

Not applicable.
